# EDCS-YOLOv8: A Lightweight YOLOv8n Variant for Ship Detection in Optical Imagery

**DOI:** 10.3390/s26216892

**Published:** 2026-10-30

**Authors:** Junxi Qiang, Yueping Peng, Yongjia Li, Wenyan Chen, Hexiang Hao, Liming Hou, Ji Wang

**Affiliations:** 1 School of Information Engineering, Engineering University of PAP, Xi’an 710086, China; carryjohn0411@163.com (J.Q.); 18212001365@163.com (W.C.); hhx1214s@163.com (H.H.); houlimingbeijing@163.com (L.H.); 15289367692@163.com (J.W.); 2 Teaching and Research Support Center, Engineering University of PAP, Xi’an 710086, China; littlewhite725@163.com

**Keywords:** ship detection, YOLOv8, EIoU loss, dynamic snake convolution, strip attention, context anchor attention, size-stratified evaluation, inland waterway surveillance

## Abstract

Ship detection in shore-based optical imagery must handle both strongly elongated vessel geometry and a severe size-dependent accuracy gap: on the SeaShips benchmark, the median annotation aspect ratio is 4.7, and a YOLOv8n baseline shows a 58-point mAP@50–95 gap between small and large ships, while, in our experiments, common small-object architectural modifications that improve small ships degrade large-ship accuracy. Motivated by these observations, in this paper, we propose EDCS-YOLOv8, a lightweight YOLOv8n variant for ship detection in optical imagery. EIoU replaces CIoU to decouple width and height in the regression of elongated boxes; a dynamic snake convolution lets the P5 backbone sample along elongated hulls instead of a rigid grid; a context anchor attention gates the SPPF output with long-range horizontal context; and a strip-gated block encodes an axis-aligned strip prior in the P3 neck to refine small-ship features. All added modules are near-identity-initialized and warm-started from COCO-pretrained weights, so training starts from the baseline mapping. Experimental results show that, on the SeaShips test set, EDCS-YOLOv8 improves overall mAP@50–95 by 0.66 points (0.48 on average across three random seeds) and medium-ship mAP by 2.00 points, with the gain positive in all three seeds and statistically significant under image-level paired bootstrap over test-set resampling (95% CI [+0.16,+0.77]); a shape-stratified analysis further shows that the improvements concentrate on the more elongated half of instances within every ship category. With 3.46M parameters and 9.0 GFLOPs—an order of magnitude lighter than ship-specific detectors in the literature—the model is suited to cost-sensitive, real-time maritime surveillance.

## 1. Introduction

Shore-based ship detection supports maritime traffic monitoring, port management, and collision-avoidance systems in coastal and inland waterways [1,2]. Automatic identification systems (AIS) and radar can suffer from coverage or resolution limitations, so camera-based detection provides a complementary source of vessel information [3]. Deep-learning-based detectors have become widely used for this task, supported by annotated benchmarks such as SeaShips [4].

Object detectors used in visual surveillance broadly include proposal-based two-stage methods [5,6,7,8,9,10,11] and one-stage detectors such as YOLO, SSD, and RetinaNet [12,13,14]. One-stage models avoid an explicit proposal stage and are therefore widely used in latency-sensitive applications; recent YOLO variants and real-time transformer detectors have further improved the accuracy–efficiency trade-off [15,16,17,18]. These characteristics make lightweight YOLO models a practical baseline for resource-constrained ship surveillance.

Recent optical ship detectors mainly improve multi-scale feature fusion [19,20], introduce attention or feature-refinement modules [21,22,23,24,25], or modify training and data-generation strategies [26]; earlier work explored CNN and lightweight YOLO variants for shore-based surveillance [27,28,29]. A parallel literature treats SAR imagery [30,31], with lightweight YOLO variants such as SHIP-YOLO [32], LH-YOLO [33], and LMSD-YOLO [34] evaluated on benchmarks such as HRSID [35] and SAR-Ship [36]. Among the studies reviewed above, evaluation is usually reported with aggregate detection metrics; size-stratified behavior and interactions between multiple added modules are reported less often.

These issues are particularly relevant on SeaShips, whose object geometry and baseline accuracy vary substantially with object scale. Statistics over the 2279 ground-truth boxes of the training split show that the annotated boxes are strongly horizontally elongated—median aspect ratio 4.74, mean 5.20; 96% of boxes exceed 2 and 45% exceed 5—and concentrated in the upper image band. On the test set, small ships (<$32^{2}$ pixels) account for 9.3% of instances but are detected at only 22.0% mAP@50–95, against 80.1% for large ships. We also evaluated several common architectural modifications intended to improve small-object detection. In our experiments, these modifications increase small-ship AP but reduce large-ship AP: adding a stride-4 P2 head improves small ships by +2.58 and degrades large ships by −5.03; inserting coordinate attention improves small ships by +0.95 at a large-ship cost of −2.77; a decoupled regression head costs −1.71 overall. In these experiments, mAP@50 stays essentially unchanged while mAP@50–95 decreases; the fact that mAP@50–95 decreases more strongly than mAP@50 is consistent with increased localization error at stricter IoU thresholds.

Based on these observations, we modify YOLOv8n at three locations. At the loss level, we replace the CIoU regression loss with EIoU [37], which penalizes width and height errors separately and adds no parameters. At the backbone, we replace the P5-stage C2f with a dynamic-snake-convolution variant [38] whose deformable sampling follows elongated structures, and fold a context anchor attention [39] into SPPF to aggregate directional context. At the neck, we insert a strip-gated block [40] with 1×19 horizontal strip kernels on the P3 branch to sharpen small-ship features. All inserted modules are near-identity-initialized and warm-started from the pretrained baseline, so training begins close to the baseline mapping. Fifteen 300-epoch trainings under a single protocol (four loss variants, five three-module combinations, and the full leave-one-out and single-module ablation grid) show that, relative to the CIoU baseline, single-module configurations change overall mAP@50–95 by +0.18 to +0.36 points and all pairwise configurations remain below the EIoU-only level, whereas the complete three-level combination reaches +0.66 and improves small-, medium-, and large-ship mAP@50–95 simultaneously. Beyond these fifteen runs, the study adds seed replicates of the three main configurations, image-level paired-bootstrap significance tests, an aspect-ratio-stratified analysis, a strip-kernel ablation, and a cross-dataset evaluation on WUTDet, for twenty-six 300-epoch trainings in total.

Our contributions are threefold. First, we quantify the aspect-ratio distribution and size-dependent baseline performance on SeaShips and use these observations to define the design targets. Second, we develop EDCS-YOLOv8 by combining EIoU with targeted backbone and neck modifications for elongated ship features, while retaining pretrained weights through near-identity initialization and warm-starting. Third, we evaluate regression losses, five candidate module combinations, and an eight-configuration ablation under a common training protocol, with results reported separately for small, medium, and large ships; the central comparisons are validated with three random seeds and image-level paired bootstrap, and an aspect-ratio-stratified analysis and a cross-dataset evaluation connect the gains to target shape and delimit their applicability.

The rest of this paper is organized as follows. Section 2 reviews ship detection, regression losses, and elongated-geometry modules. Section 3 presents the statistics-informed design and the three levels of EDCS-YOLOv8. Section 4 reports the evaluation protocol, the main results with their statistical robustness analysis, the loss and module ablations, the aspect-ratio-stratified evaluation, the cross-dataset evaluation on WUTDet, and comparisons with alternative and ship-specific detectors. Section 5 concludes.

## 2. Related Work

### 2.1. Generic Object Detection

Two-stage detectors use region proposals followed by classification and box regression [5,6,7], with later extensions improving multi-scale representation, segmentation, and high-IoU localization [8,9,10,11]; their additional proposal and refinement stages can, however, be expensive for real-time surveillance. One-stage detectors such as YOLO, SSD, and RetinaNet [12,13,14] skip the proposal stage and regress boxes directly; successive YOLO generations, efficient backbone and detector designs such as CSPNet, MobileNetV2, DAMO-YOLO, and YOLO-MS, and real-time transformer detectors [15,16,17,18,41,42,43,44] have improved the accuracy–efficiency trade-off, and drone-oriented variants add transformer prediction heads for aerial views [45,46]. Several of these detectors use channel, spatial, coordinate, or multi-scale attention as lightweight feature-refinement modules [47,48,49,50,51]; transformer-based detectors employ self-attention [52] in a different architectural form. In this work, the selected operators are linked to measured geometric properties of SeaShips, and architectural changes are kept limited to three locations.

### 2.2. Ship Detection

Optical ship detection is supported by several benchmarks: SeaShips [4] for shore-based inland-waterway surveillance, MASATI [53] for aerial views, and McShips [54] for fine-grained categorization. Recent optical ship detectors mainly improve multi-scale feature fusion [19,20], introduce attention or feature-refinement modules [21,22,23,24,25], or modify training and detection formulations, including regression losses and diffusion-based data augmentation [26,55]; earlier work explored CNN and lightweight YOLO variants for harbour and real-time surveillance [27,28,29,56]. A parallel line of research focuses on SAR imagery, where lightweight YOLO-based detectors have been evaluated on benchmarks such as HRSID and SAR-Ship [30,31,32,33,34,35,36]. These studies establish the usefulness of task-specific architectural changes, but the works reviewed here generally emphasize aggregate accuracy; size-dependent behavior and interactions between multiple added modules receive less explicit analysis. For SeaShips in particular, the prevalence of horizontally elongated boxes motivates the explicit analysis of target geometry in Section 3.1.

### 2.3. Regression Losses and Elongated-Geometry Operators

IoU-based losses are scale-invariant but blind to non-overlapping boxes; GIoU [57] adds a smallest-enclosing-box penalty; DIoU/CIoU  [58] add a normalized center-distance term and an aspect-ratio term. CIoU’s aspect-ratio penalty represents the discrepancy through a coupled arctangent term and does not penalize width and height errors separately, so it cannot distinguish boxes of equal ratio but different absolute size. EIoU  [37] decouples the penalty into independent width and height terms; SIoU [59] adds angle and shape costs; WIoU [60] reweights by a distance-attention factor; MPDIoU  [55] penalizes corner-point distances. YOLOv8 combines its IoU loss with the Distribution Focal Loss [61]. On the operator side, elongated structure has been modeled by strip pooling  [62], whose 1×k and k×1 kernels build long-range directional context; by dynamic snake convolution [38], whose deformable sampling grid snakes along tubular structures; and by strip-gated blocks in Strip R-CNN [40] and the context anchor attention of PKINet [39]. The studies reviewed above apply these operators in segmentation or aerial-detection settings; we therefore evaluate their joint use for optical ship detection under a matched training protocol.

## 3. Method

### 3.1. Statistics-Informed Design

We use statistics from the SeaShips training split to guide the changes in EDCS-YOLOv8; the test annotations are reserved for final evaluation. Across the 2279 ground-truth boxes of the training split, the aspect ratio has a median of 4.74, a mean of 5.20, and a maximum of 15; 96% of boxes exceed 2 and 45% exceed 5. Widths have a median of 606 pixels—32% of the image width—against a median height of 125 pixels, and 99.7% of boxes are wider than they are tall, so the annotated boxes are predominantly *horizontally elongated*; 98.7% of box centers lie within the upper two-thirds of the image and only 1.3% in the bottom third. By COCO area convention at the 640 training scale, small ships (<$32^{2}$) make up 7.5% of training instances, medium 44.9%, and large 47.7%; scenes are sparse (1.30 ships per image on average), and the largest class contains about five times as many instances as the smallest. Finally, the baseline’s errors are strongly scale-structured: on the test set, mAP@50–95 is 22.01 for small and 80.12 for large ships, while mAP@50 is nearly saturated (95.89 overall); the fact that the small–large gap is much larger in mAP@50–95 than in mAP@50 is consistent with localization errors at stricter IoU thresholds contributing substantially to the remaining deficit. Figure 1 summarizes the two statistics that motivate the design: the aspect-ratio distribution of the training split, and the scale-structured error profile of the baseline.

We make three targeted changes. At the loss level, EIoU replaces CIoU to penalize width and height errors separately, without changing the network architecture or adding model parameters (Section 3.2). At the P5 stage of the backbone, a dynamic snake convolution models elongated structures, and a context anchor attention folded into SPPF aggregates directional context around them (Section 3.3). At the P3 branch of the neck, a strip-gated block enlarges the horizontal receptive field for small targets (Section 3.4).

Figure 2 shows the resulting architecture. Only three positions differ from YOLOv8n—the P5 backbone C2f (A), the SPPF at the end of the backbone (B), and one insertion after the neck’s P3 C2f (C)—while every other layer, channel interface, and pretrained weight is preserved.

Every inserted module is near-identity-initialized and warm-started from the pretrained baseline (Section 3.5), so epoch zero of training closely reproduces the baseline mapping, and the added modules are subsequently optimized by gradient descent rather than by re-learning features from scratch. The full model has 3.46M parameters (+15% over the 3.01 M baseline) and 9.0 GFLOPs.

### 3.2. EIoU Localization Loss

YOLOv8 [63] supervises each assigned anchor with a Distribution Focal Loss [61] on the four box distances and an IoU-based loss on the decoded box—by default, the Complete IoU (CIoU) loss [58]. With predicted and ground-truth boxes *b*, $b^{\mathrm{gt}}$, centers c, $c^{\mathrm{gt}}$, sizes w,h and $w^{\mathrm{gt}},h^{\mathrm{gt}}$,

(1) $$L_{\mathrm{CIoU}}=1-\mathrm{IoU}+\frac{\rho^{2}\left(c,c^{\mathrm{gt}}\right)}{c^{2}}+\alpha \;v,$$

(2) $$v=\frac{4}{\pi^{2}}{\left(\arctan\frac{w^{\mathrm{gt}}}{h^{\mathrm{gt}}}-\arctan\frac{w}{h}\right)}^{2},\;\alpha =\frac{v}{1-\mathrm{IoU}+v},$$

where ρ is the Euclidean distance and *c* the diagonal of the smallest enclosing box. For elongated ships, the term *v* has two weaknesses. It constrains only the *ratio* w/h through a coupled arctangent form, so boxes with similar aspect ratios but substantially different widths and heights receive similar penalties; and as a function of the ratio alone, it does not penalize absolute size errors, which affect localization at the strict IoU thresholds that mAP@50–95 emphasizes.

EIoU [37] replaces the aspect-ratio term with separate normalized width- and height-error terms:

(3) $$L_{\mathrm{EIoU}}=1-\mathrm{IoU}+\frac{\rho^{2}\left(c,c^{\mathrm{gt}}\right)}{c^{2}}+\frac{{\left(w-w^{\mathrm{gt}}\right)}^{2}}{C_{w}^{2}}+\frac{{\left(h-h^{\mathrm{gt}}\right)}^{2}}{C_{h}^{2}}.$$

Width and height errors are thus penalized directly and separately with scale-invariant normalization, so the loss can steer a box toward the correct size even when its center is already aligned. Because only the regression objective is changed, EIoU introduces no additional layer, branch, or model parameter: it changes the regression gradients but leaves the detector architecture unchanged.

### 3.3. Backbone: Elongated-Geometry Representation

#### 3.3.1. Dynamic Snake Convolution on P5

P5 is the lowest-resolution feature level and is primarily responsible for representing larger objects in the detection hierarchy. We replace the last backbone C2f with a variant whose bottleneck convolution is a dynamic snake convolution [38] (DSConv; Figure 3a). A DSConv maintains a linear k×1 or 1×k sampling grid whose every offset is selected jointly with its neighbors through a dedicated offset branch: an offset field predicted from the input is passed through tanh, scaled by an extent hyperparameter, and applied by bilinear sampling, so the kernel *snakes* along the elongated structure instead of sampling a rigid rectangle. Unlike unconstrained deformable sampling, DSConv imposes continuity along a single axis, providing an inductive bias for elongated structures. Our configuration uses a kernel size of k=3 with an extent hyperparameter (scope) of 1; the offset branch is a 3×3 convolution producing 2k channels, batch-normalized and passed through tanh before bilinear sampling, and the sampled features are convolved by the axis-specific k×1 or 1×k kernel, followed by group normalization and a SiLU activation. For stable warm-starting, we make two implementation changes: the offset branch is zero-initialized, so at epoch zero the snake degenerates to a straight standard sampling (avoiding random deformation at initialization); and a projection branch is initialized to route the original convolution output identically, so the module closely reproduces the pretrained C2f bottleneck mapping at initialization. The pretrained bottleneck weights are copied into the main branch by warm-start.

#### 3.3.2. Context Anchor Attention in SPPF

The context anchor attention (CAA) of PKINet [39] (Figure 3b) captures long-range directional context in strip form: the input is average-pooled by a 1×11 kernel along the horizontal axis and then along the vertical axis, the pooled descriptor is embedded by a 1×1 convolution, and a sigmoid gate modulates the input feature map. The band-shaped receptive field is intended to emphasize directional context consistent with the predominantly horizontal elongation observed in the dataset. To keep the channel layout at the insertion point unchanged, we fold CAA into SPPF: SPPF first aggregates multi-scale max-pooled context as usual, and CAA then gates its output, preserving the 256-channel interface so that the surrounding architecture and pretrained weights are unaffected. CAA’s gate convolution is initialized with $N(0,10^{-3})$ weights and a batch-norm bias of 4, giving σ(silu(4))≈0.98: the module starts as a near-identity pass-through that retains nonzero gradients at initialization (dσ/dx≈0.019).

### 3.4. Neck: Strip-Gated Refinement on P3

P3 is the highest-resolution detection feature and is therefore particularly relevant to small objects. After the P3 C2f of the neck we insert a strip-gated block [40,62] (Figure 3c). The block computes an attention residual $x+\lambda \;\sigma \;\left(f_{1\times 1}\;S\left(x\right)\right)⊙x$, where S(·) is a depthwise cascade of a 5×5 kernel followed by a 1×19 and a 19×1 strip kernel, $f_{1\times 1}$ is a pointwise convolution, σ is a sigmoid, and the layer scale $\lambda =10^{-2}$ makes the block near-identity at initialization. The 1×19 kernel provides a long horizontal receptive field on P3, consistent with the strong horizontal elongation observed in the ship boxes, and together with its vertical counterpart, it encodes the axis-aligned strip prior directly into the P3 refinement path. The block is channel-preserving (64→64), so it is inserted as a new layer with index remapping during warm-start (Section 3.5).

### 3.5. Near-Identity Initialization and Warm-Start Protocol

All twenty-six 300-epoch runs share the same initialization and warm-start protocol. We initialize the added modules close to the identity mapping using a positive gate bias together with small random weights: the bias (typically 4–8, before sigmoid/silu) passes the input through nearly unchanged, while the $N(0,10^{-3})$ weights keep a small but nonzero gradient path. In preliminary experiments, exact zero initialization blocked gradient propagation through the added branch and was therefore not used. Warm-start then transfers the COCO-pretrained YOLOv8n weights (official Ultralytics release): layers whose names match are copied directly; layers shifted by the insertion of the neck block are remapped by index offset; and the snake convolution’s main branch inherits the pretrained bottleneck weights. A loading report generated by the released warm-start script records the status of every tensor of the final model: 204 tensors are copied by exact name match and 145 by index remapping after the neck insertion—2.86 M parameters in total, 82.7% of the model—while the 88 tensors of the inserted modules (0.60 M) start from the near-identity initialization; the remaining 151 checkpoint tensors all belong to the 80-class COCO detection head and remain unused, as expected (Appendix B). Consequently, the enhanced model closely reproduces the baseline mapping at initialization, and the ablation of Section 4.7 measures what training *adds* to that starting point. A preliminary comparison also showed that a cold-started deformable convolution variant reduced large-ship mAP@50–95 by 0.70 points, whereas the same operator warm-started improved it by 0.20 points.

## 4. Experiments

### 4.1. Dataset

SeaShips [4] contains 7000 precisely annotated optical images (1920×1080) captured by shore-based cameras in the Hengqin district of Zhuhai, spanning six classes: ore carrier, bulk cargo carrier, general cargo ship, container ship, fishing boat, and passenger ship. We follow the standard split of 1750 training, 1750 validation, and 3500 test images and report all results on the test set. The design statistics of Section 3.1 are computed on the training split (2279 ground-truth boxes); the test annotations are used only for final evaluation.

### 4.2. Implementation Details

All models are trained with Ultralytics YOLOv8 [63] (v8.4.121, PyTorch 2.5.1) on a single RTX 4060 Laptop GPU (8 GB). Every configuration uses the identical recipe: COCO-pretrained YOLOv8n weights (official Ultralytics release), 300 epochs, batch size 16, input size 640×640, SGD with the default schedule (initial learning rate 0.01, momentum 0.937, weight decay $5\times 10^{-4}$), default mosaic augmentation, seed 0, and deterministic training. Inserted modules follow the near-identity-initialization and warm-start protocol of Section 3.5. The complete study comprises twenty-six 300-epoch trainings: the CIoU baseline, three alternative losses (EIoU/SIoU/WIoU), five three-module combinations, the single-module and leave-one-out grid around the winning combination, seed replicates (seeds 1 and 42) of the three main configurations, a loss–architecture factor completion, a strip-kernel ablation, and three runs on the WUTDet Part A dataset (Section 4.12). Inference cost is measured on the same GPU (batch size 1, FP32, 640 input, 60 test images after 10 warm-up passes); we report the model-forward latency, which excludes pre- and post-processing and is stable to ±0.01 ms across images. End-to-end system latency is not evaluated. The three main configurations (CIoU baseline, EIoU-only, and the full model) are trained with three seeds each; all remaining entries are single runs at seed 0. Several reported differences are below one mAP point, and Section 4.5 quantifies the training and sampling variance of the central comparisons.

### 4.3. Metrics and Size-Stratified Protocol

We report precision, recall, and mean average precision at IoU 0.5 (mAP@50) and averaged over IoU 0.50–0.95 (mAP@50–95). To examine scale-dependent behavior that is not visible in aggregate mAP, every evaluation is accompanied by a *size-stratified* decomposition in the COCO convention [64]: ground-truth boxes are bucketed by area at the 640 scale into small (<$32^{2}$), medium ($32^{2}$–$96^{2}$), and large (>$96^{2}$), and mAP is computed per bucket by per-class greedy matching over the IoU ladder with 101-point interpolation, using streaming inference at confidence 0.001 with NMS at IoU 0.7. The identical protocol, implemented in a single evaluation script, is applied to all trained models, so every table in this paper is directly comparable column by column. Three accounting details matter for reproduction. First, matching is greedy per class and per IoU threshold; each detection may claim at most one ground truth and vice versa, and detections are capped at 300 per image (the library default). Second, across size buckets, a detection matched to a ground truth outside the current bucket is excluded from that bucket (neither true nor false positive), whereas unmatched detections count as false positives in every bucket; this is a stricter convention than the native area-range statistics of the reference COCO evaluator, which additionally mark unmatched detections whose own predicted-box area falls outside the range as ignored. Third, on matched settings our implementation reproduces the aggregate output of pycocotools exactly (75.12% baseline mAP@50–95 over all sizes); the stratified small- and medium-ship values consequently read lower than COCOeval area-range statistics by construction, and all model-to-model comparisons use identical semantics. The complete evaluation and analysis code, including the size-stratified protocol, the bootstrap analysis, and the warm-start scripts, is publicly available (see the Data Availability Statement).

### 4.4. Main Results

Table 1 summarizes the three stages of the design. In the seed-0 runs, EIoU alone improves the observed overall mAP@50–95 by +0.34 without any architectural change; Section 4.5 shows that this standalone margin lies within training variance, whereas the improvements of the full model are statistically validated. The full configuration reaches 75.78% mAP@50–95, compared with 75.12% for CIoU and 75.46% for EIoU alone. Relative to CIoU, the observed changes for small, medium, and large ships are +1.71, +2.00, and +0.03 points, respectively; across seeds, the medium-ship gain is the most stable (the full model has the highest medium mAP of the three configurations in every seed), while the small bucket, which contains 435 test instances, is the noisiest (Section 4.5). This modification increases the parameter count from 3.01M to 3.46M (+15%), GFLOPs from 8.1 to 9.0 (+11%), and model-forward latency from 1.67 to 2.41 ms; end-to-end system latency is not evaluated. The full model has slightly lower mAP@50 than EIoU alone (96.17 vs. 96.45) but higher mAP@50–95 (75.78 vs. 75.46), a pattern consistent with differences emerging mainly at stricter IoU thresholds.

### 4.5. Statistical Robustness of the Main Comparisons

Because the central differences are smaller than one mAP point, we quantify their statistical robustness along two axes: training variability and test-set composition.

**Seed replicates.** We retrained the CIoU baseline, the EIoU-only variant, and the full model with seeds 1 and 42 in addition to seed 0 (Table 2). The paired differences are positive in all three seeds for EDCS−CIoU (+0.66, +0.03, +0.74; mean +0.48) and for EDCS−EIoU (+0.32, +0.24, +0.75; mean +0.44), whereas EIoU−CIoU changes sign across seeds (+0.34, −0.21, −0.01; mean +0.04). The medium bucket is the most stable beneficiary: in every seed the full model attains the highest medium-ship mAP of the three configurations, with per-seed gains of +0.44 to +2.00 over the baseline. The small bucket is the noisiest—its 435 test instances produce a baseline standard deviation of ±1.31—so single-seed small-ship differences should be read cautiously; the full model has the smallest small-ship spread of the three configurations (±0.26).

**Paired bootstrap.** To quantify the uncertainty associated with the composition of the test set, we perform B=5000 bootstrap iterations; in each iteration, one resample of the 3500 test images (with replacement) is drawn and evaluated for all nine seed-matched models, and the mean of the three per-seed differences is recorded [65] (Table 3). Because every seed is evaluated on the same resampled image set, the correlation among seed-matched evaluations is preserved. The EDCS−CIoU bootstrap mean is +0.46 with a percentile-based 95% confidence interval of [+0.16,+0.77] and a proportion of positive differences of 0.999; EDCS−EIoU is +0.43 with [+0.12,+0.72] and 0.996. Both intervals exclude zero. The EIoU−CIoU difference (+0.04, [−0.25,+0.31]) does not. The bootstrap means differ slightly from the full-test-set three-seed means of Table 2 (+0.48 and +0.44) because mAP is a nonlinear statistic of the resampled set. We note the inferential scope of these intervals: they characterize test-image-sampling uncertainty *conditional on the three trained models per configuration*; training variability is reported separately by the per-seed differences above, and with three replicates we do not attempt a joint hierarchical inference over seeds.

**Consequences for our claims.** (i) The complete configuration improves over both the CIoU baseline and the EIoU-only variant with margins that survive seed and resampling variation. (ii) The standalone loss replacement is not a statistically reliable source of aggregate improvement on this benchmark; EIoU is therefore retained as a validation-selected, parameter-free component of the evaluated design, and no independently established contribution is claimed for it (Section 4.6 and Section 4.7). (iii) We avoid rank-order claims between configurations whose differences are below this noise floor, such as the top two module groups of Section 4.8, which differ by +0.04 (Table 4).

### 4.6. Loss-Level Ablation

Table 5 compares CIoU, EIoU, SIoU, and WIoU using the same architecture and training protocol. All three alternatives yield slightly higher observed overall mAP@50–95 than CIoU in these runs, and none reduces large-ship mAP by more than 0.41—the structural trade-off of Section 4.10 does not occur at the loss level. The losses differ in where their gains appear: WIoU produces the largest small-ship improvement (+2.54) with little medium gain, whereas SIoU yields the highest medium-ship result among the tested losses but is nearly neutral on small. We select EIoU because it gives the highest observed overall and validation mAP@50–95 among the tested losses while improving both small- and medium-ship AP relative to CIoU; the validation-split record reproduces this ordering (Appendix A). The seed and bootstrap analyses of Section 4.5 qualify this selection: the standalone EIoU-over-CIoU margin (+0.34 in the seed-0 run) averages +0.04 across seeds with a confidence interval containing zero, so EIoU should not be read as an independent source of aggregate improvement. Its within-architecture margin in the single factor run is +0.15 (Table 6); this observation is not independently replicated, and no synergistic interaction with the structural modules is claimed. Figure 4 visualizes the four-loss comparison.

Table 6 separates the two factors. At fixed loss, the architecture contributes +0.51 (CIoU) and +0.32 (EIoU); at fixed architecture, the loss contributes +0.34 (baseline) and +0.15 (EDCS). The geometric modules alone (EDCS architecture with CIoU) already recover most of the improvement, with +2.20 small, +1.57 medium, and −0.10 large relative to the CIoU baseline. Together with the multi-seed analysis (Section 4.5), the load-bearing part of the improvement is associated with the geometric architecture; the single-run factor grid does not support claims about the loss–architecture interaction.

**Figure 4:**
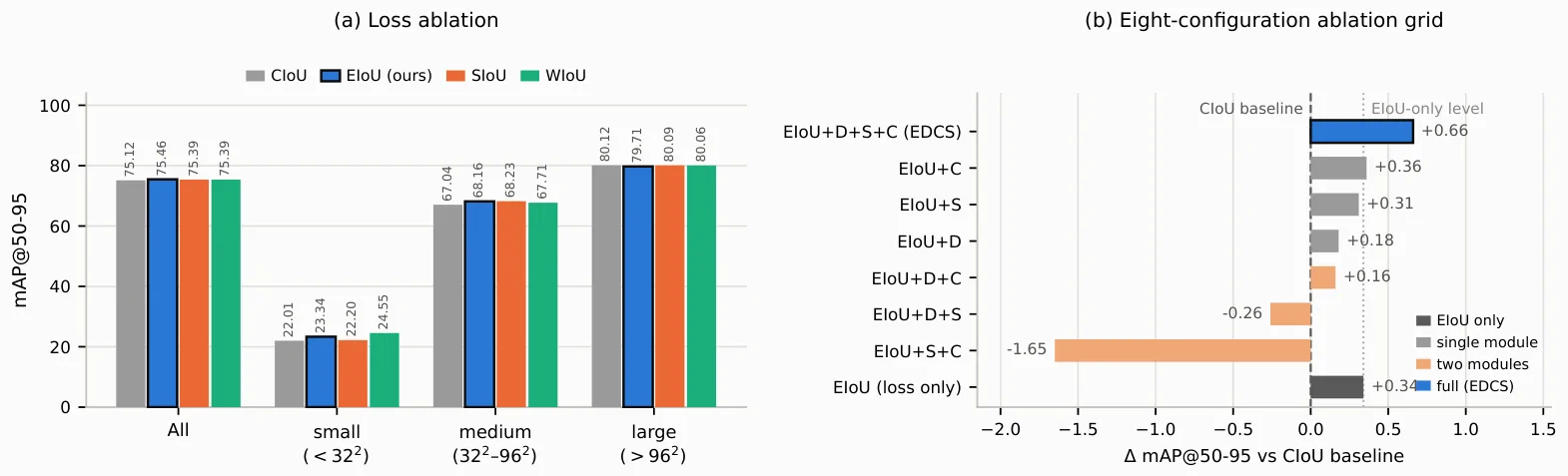
(**a**) Size-stratified mAP@50–95 of the four regression losses: EIoU improves small and medium ships over CIoU while leaving large ships essentially unchanged. (**b**) The eight-configuration ablation grid of Table 7 as deltas of All mAP@50–95 relative to the CIoU baseline (zero line); the dotted line marks the EIoU-only level (+0.34). Single-module and pairwise variants stay at or below that level, and only the complete configuration exceeds it (+0.66). Δ denotes the change relative to the CIoU baseline.

### 4.7. Module Ablation and Interaction Analysis

Table 7 reports the complete single- and pair-module grid around the full model, with every cell trained for 300 epochs under the identical protocol. Three observations follow.

**Overall mAP across module combinations.** Relative to the CIoU baseline, the three single-module rows gain +0.18 to +0.36 points—the level of the EIoU-only gain (+0.34)—while the pairwise rows span −1.65 to +0.16 and never exceed that level. The complete D+S+C configuration reaches +0.66 over CIoU (versus +0.34 for EIoU alone), with small +1.71, medium +2.00, and large +0.03; relative to the EIoU-only row, it is +0.32. Relative to the full model, removing D, C, or S reduces overall mAP@50–95 by 2.31, 0.92, and 0.50 points, respectively. The largest contrast is the pair S+C: it reaches 73.47, 1.65 points below even the CIoU baseline, whereas D+S+C reaches 75.78; this contrast is consistent with an interaction between D and the S+C combination in the tested configuration. Figure 4b shows the same structure on the CIoU-referenced scale: single-module bars stay within 0.16 points of the EIoU-only level (+0.34), all pairwise bars are below it, and only the complete configuration exceeds it (+0.66).

**Size-dependent effects.** Among the single-module variants, S gives the highest small-ship mAP@50–95 (25.47, +2.13 over EIoU), while C gives the highest large-ship result (80.08, +0.37). D alone falls within 0.16 points of the EIoU-only row in overall mAP@50–95 (75.30 vs. 75.46) but produces the largest reduction when removed from the full model (−2.31).

**Interaction structure.** Measured against the EIoU-only row, the three single-module effects (−0.16, −0.03, +0.02) sum to −0.17, yet the complete configuration is +0.32 above that row. The seed replicates and bootstrap analysis (Section 4.5) show that this full-configuration margin is not a seed artifact: EDCS exceeds the EIoU-only model in all three seeds (mean +0.44) with a pooled 95% confidence interval of [+0.12,+0.72], whereas the single-module and pairwise rows remain single runs whose mutual ordering is within noise. One possible reading is configuration-dependent interaction among the structural modules: the deformable sampling of the snake convolution may supply an elongated-geometry representation that the strip-gated refinement on P3 and the directional context attention in SPPF then exploit, leaving each near-identity perturbation alone too weak to move aggregate mAP. The present single-run ablations do not isolate these mechanisms, and the degradation of partial combinations (the S+C pair, itself a single run, reaches −1.65) is reported as an empirical observation rather than as evidence of a specific interference mechanism. The aspect-ratio-stratified analysis of Section 4.9 is consistent with this reading, which we present as an interpretation rather than a proven decomposition.

### 4.8. Combination Search

Before the ablation grid, we screened five three-module combinations chosen along independent design axes (Table 4), each trained and evaluated under the same protocol; screening decisions were based on the validation split (Appendix A), and Table 4 reports test numbers for the resulting configurations. Three groups satisfy the predefined acceptance criterion; two do not. In this five-configuration search, the three configurations meeting the criterion all include a *horizontal strip kernel* (G1: 1×19 strip gating and 1×11 anchor attention; G2: 1×19 strip-convolution GLU and 1×11 separable large-kernel attention; G4: 1×19 strip gating at the neck), whereas the two configurations that do not satisfy it (lightweight attention; a cross-axis combination without strip priors) lack this component. The large-ship result of the attention-only group (G3, −1.17 vs. the EIoU row of Table 4) is consistent with the regression measured for plain attention modules in Section 4.10. This pattern is consistent with the hypothesis that horizontal strip priors are useful for the strongly elongated objects in SeaShips (median aspect ratio 4.7); because only five combinations were evaluated, we do not interpret it as a general predictor of module effectiveness.

### 4.9. Aspect-Ratio-Stratified Evaluation

The size buckets of Section 4.3 measure scale, whereas the design of Section 3.1 targets shape. To connect the improvements to elongation directly, we stratify the test set by aspect ratio (AR =w/h of the ground-truth box). Values in this subsection are computed on instance subsets with the size-bucket convention of Section 4.3: a detection matched to a ground truth outside the subset is excluded from that subset (neither true nor false positive), while unmatched detections count as false positives. They are not comparable in absolute terms with the size-bucket tables, and models are compared only within the same subset.

**AR terciles.**Table 8 splits the 4683 test instances into terciles (boundaries 3.92 and 5.66). The EDCS−CIoU difference rises monotonically with aspect ratio: −0.28 in the low tercile, +0.83 in the middle, and +3.34 in the high tercile. Two confounds, however, prevent reading this as a pure shape effect: the class composition differs across terciles (the low tercile is dominated by general cargo ships, 647 of 1561; the high tercile by fishing boats, 834 of 1561), and mean box area grows with AR (10,864→15,532 px^2^).

**Within-class control.** To remove the class confound, we split *each class* at its own AR median and compare the halves (Table 9). Class identity is then held fixed within each row; the halves need not be scale-matched, and their mean areas are listed to keep this residual difference visible. Within every one of the six classes, EDCS improves over the CIoU baseline on the high-AR half (from +0.92 to +1.86; mean +1.53, six of six positive), whereas the low-AR half is essentially unchanged (from −0.86 to +1.08; mean +0.25, two of six slightly negative). The gains of the geometric design therefore concentrate on the elongated instances of each category without a measurable penalty on the compact instances of the same categories.

**Accounting sensitivity.** Subset results depend on how detections that match ground truths outside the subset are handled. Under the exclusion rule used above, the low tercile is essentially neutral. A stricter rule that instead counts such detections as false positives in every subset reads the low tercile at −6.96 (mean −6.44 over the within-class low halves), because detectors that find more ships outside the subset are penalized inside it; a COCO-style rule that additionally ignores unmatched detections overlapping outside ground truths at IoU ≥0.5 flattens the gradient further. The high-AR gains stay positive under all three rules (+1.03, +3.34, and +0.61 for the high tercile, respectively), so we base conclusions only on them and do not assert a compact-instance penalty.

**Strip-kernel size.** The 1×19/19×1 kernels of the neck block follow the same reasoning: the median ground-truth box is 606 px wide at the 1920×1080 acquisition scale, i.e., about 202 px at the 640 training scale, and a 1×19 kernel on the stride-8 P3 level spans 19×8=152 px, closer to that length than the 88 px spanned by 1×11; strip kernels of comparable length are standard in strip pooling [40,62]. Empirically, replacing the 1×19/19×1 kernels with 1×11/11×1 (one 300-epoch run, seed 0) lowers every size bucket: All 75.37 vs. 75.78, small 23.15 vs. 23.72, medium 68.74 vs. 69.04, and large 79.41 vs. 80.15; every difference favors the 1×19 kernels.

### 4.10. Comparison with Alternative Structural Modifications

Table 10 summarizes the additional structural modifications evaluated during model development, each trained under the same protocol. In the tested alternatives, improvements for small ships are accompanied by reductions of 1.6–5.0 points for large ships. These results motivate us to limit architectural perturbations to minimal, targeted insertions (near-identity-initialized, warm-started, channel-preserving) and to place part of the localization adjustment in the regression loss, which adds no model parameters. Among the configurations in Table 10, EDCS-YOLOv8 is the only one with positive observed deltas for both the small and large groups.

### 4.11. Comparison with Ship-Specific Detectors

Table 11 lists reported results from several ship-specific detectors for contextual reference. These results are not directly comparable because the studies use different data splits, model scales, input settings, and hardware. We therefore do not use Table 11 to rank accuracy or inference speed; quantitative claims about the improvement of EDCS-YOLOv8 are based on the protocol-matched comparisons in Table 1, Table 2, Table 3, Table 4, Table 5, Table 6, Table 7, Table 8, Table 9 and Table 10. Parameter count can nevertheless be compared directly: EDCS-YOLOv8 contains 3.46M parameters, substantially fewer than the systems listed in Table 11.

Two considerations frame the deployment trade-off. First, the reference frame for “lightweight”: the +15% parameter and +0.74 ms forward cost is measured against the YOLOv8n *baseline*, whereas against the ship-specific detectors of Table 11 the model is an order of magnitude lighter (3.46 M vs. 29.8–42.5 M parameters) while sustaining ≈415 FPS on a laptop GPU; in absolute terms, both the baseline and the full model are real-time for typical surveillance frame rates. Second, when the additional cost is justified: the statistically validated gains concentrate on medium and elongated small vessels at strict localization thresholds (Section 4.5 and Section 4.9). Scenes dominated by compact or large vessels at generous IoU requirements, or deployments with hard latency or power budgets, are adequately served by the baseline. The loss-only variant is a zero-parameter, near-zero-latency option, but its standalone gain is within noise (Section 4.5); its reliable benefit appears only as part of the full configuration.

### 4.12. Cross-Dataset Evaluation on WUTDet

The aspect-ratio analysis of Section 4.9 implies that the gains of the geometric design depend on the shape signature of the target data rather than on ship detection in general. To test this prediction on a second dataset, we train and evaluate the same three configurations on WUTDet [66], a shipborne electro-optical dataset collected on board a supply vessel over three months. The publicly released Part A subset contains 5025 single-class *ship* annotations (1920×1080, 81%; 2560×1440, 19%); we train on its 4020-image train split with the identical 300-epoch recipe (COCO warm start, seed 0) and reserve the 1005-image val split as an untouched test set (3770 instances).

WUTDet Part A is small-object dominated but geometrically opposite to SeaShips: 78.7% of its test instances are small at the 640 scale (against 9.3% on SeaShips), while the aspect-ratio signature is compact rather than elongated—median AR 1.38 (SeaShips 4.74), only 0.2% of instances above AR 5 (45% on SeaShips), and 67.5% of boxes wider than tall (99.7% on SeaShips). WUTDet Part A therefore provides a targeted test case: a dataset where ships are abundant but elongated structure is not.

Table 12 shows the outcome: the three configurations lie within single-seed noise of one another, and the SeaShips advantage of the full model does not transfer. We report this as a boundary of applicability. The geometric modules were selected for the elongated signature measured on SeaShips (Section 3.1); the substantially lower prevalence of elongated targets on WUTDet is consistent with the absence of a clear advantage, in line with the shape dependence of Section 4.9, where the gains concentrate on the elongated instances of each class. The result supports the statistics-driven design principle underlying this work: elongated-geometry modifications should be expected to help where elongated targets exist, and a shape-signature check of the target dataset is the appropriate screening step before applying them. WUTDet also differs from SeaShips in viewpoint, instance density, and class taxonomy, and the comparison is single-seed; the experiment therefore delimits where the advantage was observed rather than isolating aspect ratio as the sole cause. Whether the modules transfer to other elongated-signature domains, such as SAR or infrared ship imagery, remains open.

### 4.13. Per-Class Results and Error Analysis

Table 13 decomposes the aggregate gain by class. EDCS attains the best per-class AP in four of the six categories and improves over the CIoU baseline in five of six. The only exception, general cargo ships (−0.24), is among the most compact classes (median AR 3.8), consistent with the shape dependence of Section 4.9; the largest per-class gains occur on the strongly elongated ore carriers and fishing boats. The aggregate improvement is therefore shared across the category spectrum rather than carried by a single class.

Figure 5 shows two examples of distant, elongated small ships missed by the CIoU baseline and detected by EDCS-YOLOv8. These targets are represented on the high-resolution P3 branch, where the strip-gated refinement is applied. In the same crops, the surrounding large-ship detections appear visually similar for the two models. These examples are qualitative and are not used as evidence beyond the quantitative results in Table 1.

To characterize the remaining errors, we analyze the full-model predictions at confidence ≥0.5 on the test set. Of the 4683 ground-truth instances, 90 (1.9%) have no same-class detection at IoU ≥0.5. Classified by the best overlapping prediction of *any* class, 36 are localization-type misses (a same-class prediction overlaps with IoU 0.1–0.5: the object is seen but the box is loose), 39 are classification-driven misses (a prediction covers the target at IoU ≥0.5 but carries a different class label, so no true positive is credited), 5 are partial (any-class overlap 0.1–0.5), and only 10 are truly unseen (no prediction of any class reaches IoU 0.1). Misses are twice as frequent on compact instances as on elongated ones (60 with AR <5 vs. 30 with AR ≥5), consistent with the design’s emphasis on elongated geometry. False positives (208 in total) split into background confusion (84), classification errors (73), and localization errors (51); classification confusion is thus the largest single miss mechanism (39 of 90) and the second-largest false-positive category. A wrong-class detection is counted once as a classification-driven miss (ground-truth view) and once as a classification false positive (prediction view); the two ledgers are different views of the same events, not independent error counts. Figure 6 shows one representative of each failure mode: a localization-type miss, a classification-driven miss, a classification false positive, and a localization false positive.

## 5. Conclusions

This paper presented EDCS-YOLOv8, a lightweight modification of YOLOv8n for optical ship detection that combines a modified regression objective with targeted backbone and neck refinements motivated by the elongated geometry and scale-dependent performance observed on SeaShips. The added modules use near-identity initialization and warm-starting from the pretrained baseline.

On the SeaShips test set, the full model reaches 75.78% mAP@50–95, compared with 75.12% for the CIoU baseline and 75.46% for EIoU alone (seed 0), at 3.46M parameters and 2.41 ms model-forward latency. Across three random seeds, the paired gain over the CIoU baseline averages +0.48 and is statistically significant under image-level paired bootstrap (95% CI [+0.16,+0.77]); the standalone loss replacement is within training noise, so the validated contribution is the complete configuration. The largest size-specific change relative to CIoU occurs for medium ships (+2.00), and this bucket is also the most stable across seeds. The aspect-ratio-stratified analysis shows that within every ship class the gains concentrate on the more elongated half of instances (mean +1.53 points, six of six classes) while the more compact half is essentially unchanged, and the cross-dataset evaluation on WUTDet—a small-object dataset without an elongated signature—shows that the advantage does not transfer there, delimiting the applicability of the geometric design. In the five-group search, the three configurations meeting the acceptance criterion all contain horizontal strip kernels, and the observed module effects are non-additive across the tested configurations.

This study has limitations. (1) Conclusions rest on two optical shipborne/shore-based benchmarks; SAR and infrared domains remain untested. (2) The aggregate improvement is modest (+0.66 points in the seed-0 protocol), and seed replicates cover only the three main configurations; the rest of the ablation grid remains single-seed. (3) Latency was measured as the model forward pass on a laptop GPU; end-to-end latency and deployment on embedded edge hardware are unverified. (4) The aspect-ratio-stratified analysis is sensitive to how cross-subset detections are accounted for; we therefore restrict our claims to the elongated-side gains, which remain positive under all tested accounting rules. Future work will pursue four directions: distillation and pruning to recover the baseline’s latency while keeping the size-group gains; scale-adaptive weighting of the regression penalties, suggested by the differing size-group behavior of EIoU, SIoU, and WIoU; extension to video surveillance with temporal consistency, a relevant deployment scenario for inland-waterway monitoring; and a shape-signature screening procedure that decides, from dataset statistics alone, when elongated-geometry modules are appropriate.

## Figures and Tables

**Figure 1:**
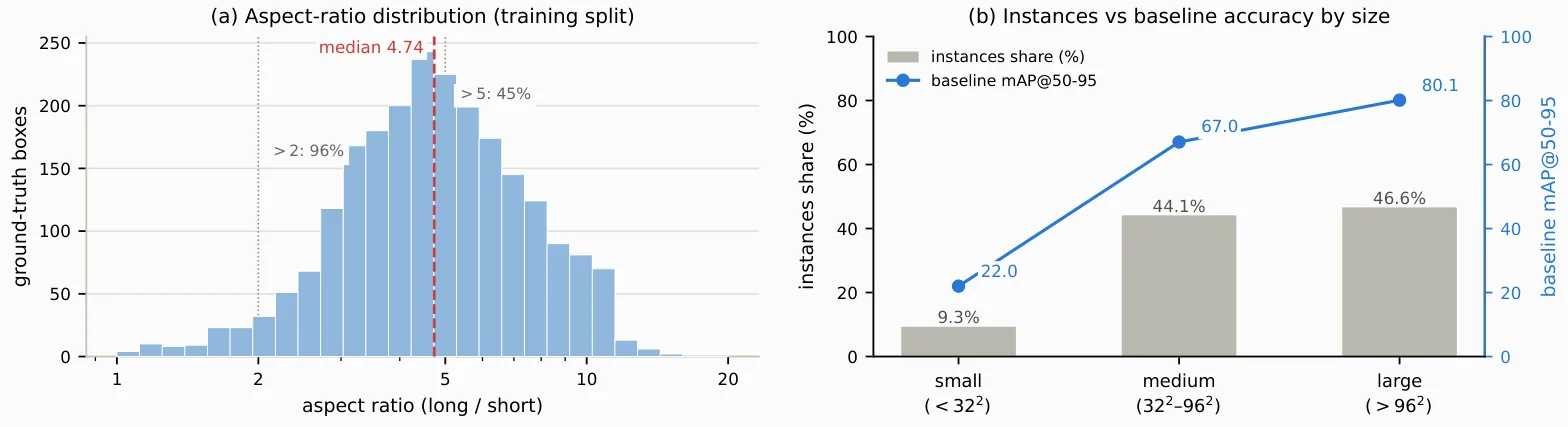
Dataset statistics that motivate EDCS-YOLOv8. (**a**) Aspect-ratio distribution of the 2279 ground-truth boxes in the SeaShips *training* split (median 4.74; 96% of boxes exceed 2 and 45% exceed 5). (**b**) Per-size-group instance share of the *test* split against the CIoU baseline’s size-stratified mAP@50–95: small ships carry 9.3% of test instances but reach only 22.01 mAP@50–95, versus 80.12 for large ships.

**Figure 2:**
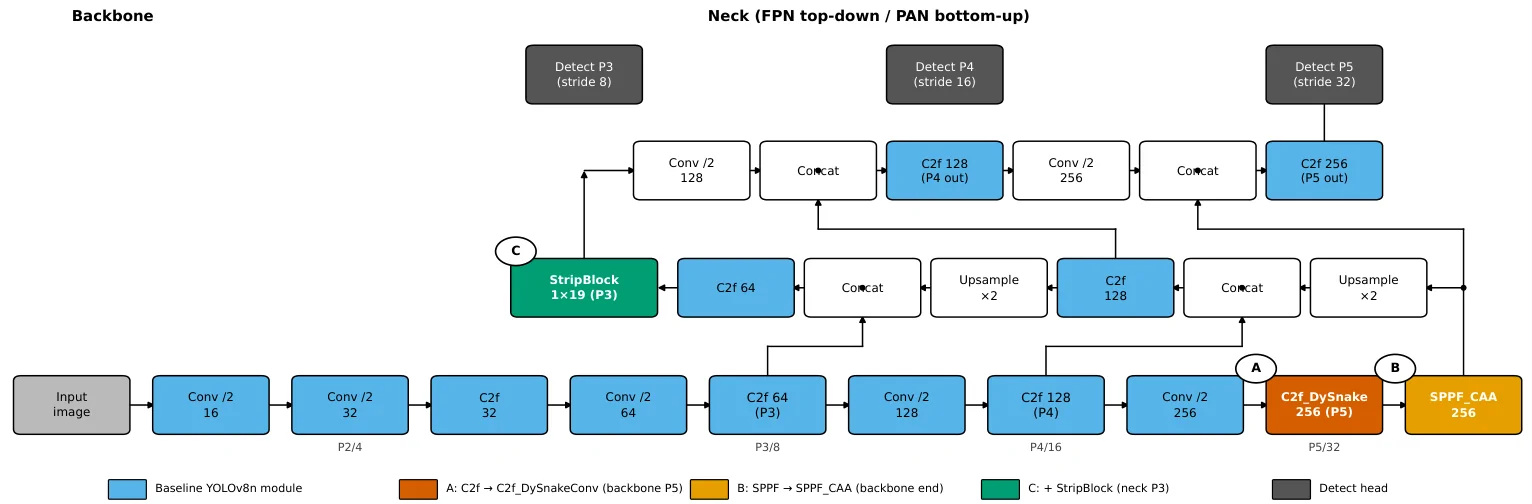
The EDCS-YOLOv8 architecture. Relative to the YOLOv8n baseline (light blue), only three positions differ: **A** the P5 backbone C2f becomes C2f_DySnakeConv (vermilion), **B** the SPPF at the end of the backbone becomes SPPF_CAA (orange), and **C** a strip-gated block is inserted after the neck’s P3 C2f (green). Stride markers P2–P5 indicate feature resolutions; **A**/**B**/**C** correspond to the three design levels of Section 3.1.

**Figure 3:**
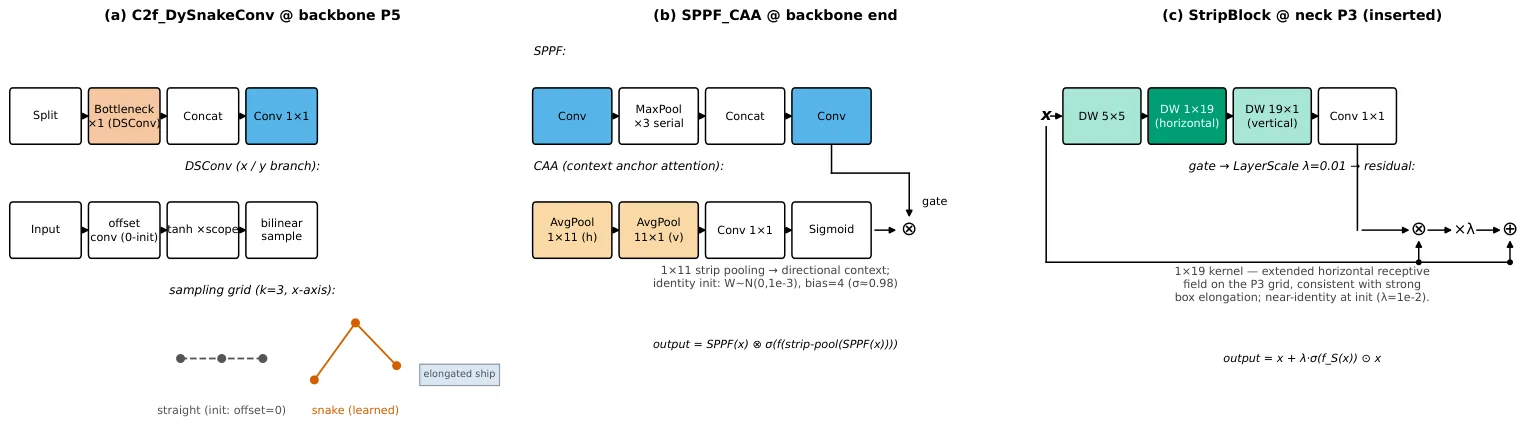
The three inserted modules. (**a**) C2f_DySnakeConv: the bottleneck convolution of the P5 C2f becomes a dynamic snake convolution whose zero-initialized offset branch lets the sampling grid snake along elongated hulls instead of covering a rigid rectangle. (**b**) SPPF_CAA: a context anchor attention with 1×11/11×1 strip average pooling gates the SPPF output; the gate is near-identity-initialized ($N(0,10^{-3})$ weights, bias 4). (**c**) StripBlock: a depthwise 5×5→1×19→19×1 strip cascade computes a sigmoid gate, scaled by $\lambda =10^{-2}$ and added residually: $x+\lambda \;\sigma \left(f_{1\times 1}S\left(x\right)\right)⊙x$. Arrows indicate the direction of data flow; ⊕, ⊗, and ⊙ denote element-wise addition, multiplication, and gated multiplication, respectively.

**Figure 5:**
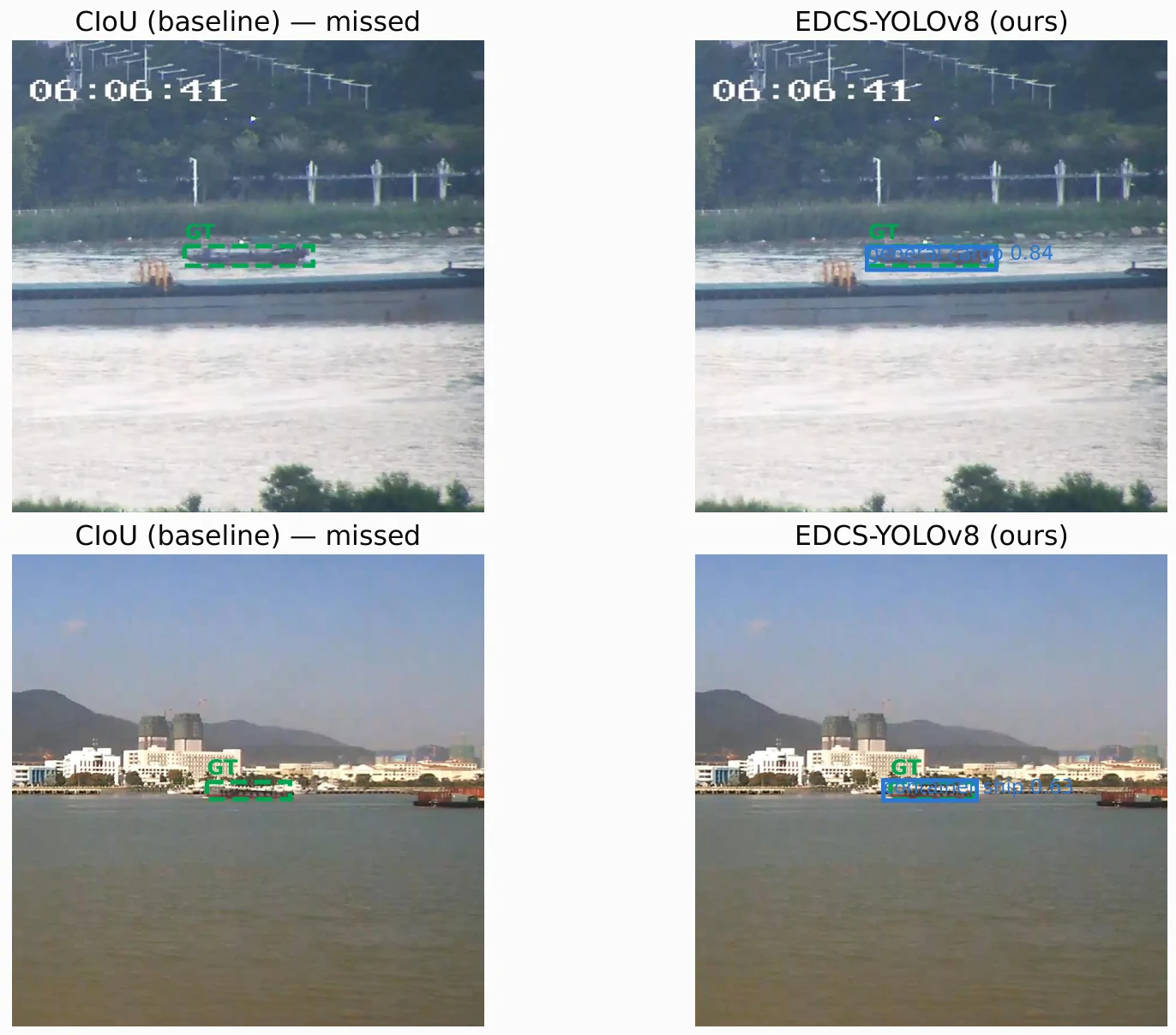
Qualitative examples (test set, cropped around each target). Dashed green boxes are ground truth. Left: the CIoU baseline produces no same-class detection at IoU ≥ 0.5 for the small ship. Right: EDCS-YOLOv8 detects it with high confidence.

**Figure 6:**
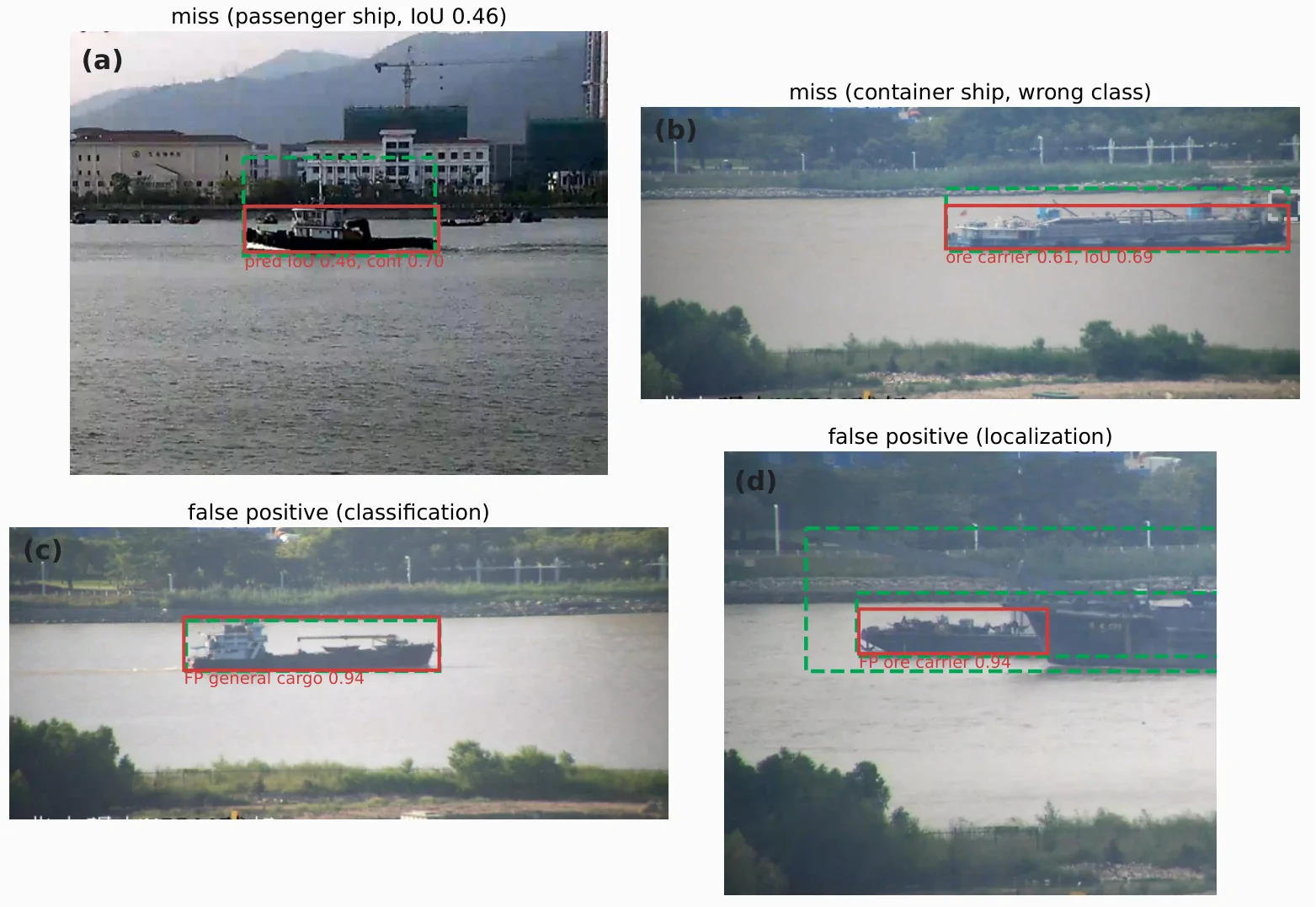
Remaining failure modes of EDCS-YOLOv8 on the test set. Green dashed: ground truth; light-gray dashed (**a**,**b**): further annotated ships in the same crop; red: model prediction. (**a**) A localization-type miss: the ship is detected, but the most confident same-class box overlaps just below the IoU 0.5 threshold. (**b**) A classification-driven miss: a high-confidence *ore carrier* box covers the target at IoU 0.69, but no container-ship true positive is credited. (**c**) A classification false positive: a high-overlap detection assigned to the wrong class. (**d**) A localization false positive: correct class, IoU below 0.5.

**Table 1:** Main results on the SeaShips test set (size-stratified mAP@50–95, %). Inference latency is the model forward pass (batch 1, FP32, 640 input, RTX 4060 Laptop). The best value in each column is shown in bold.

Method	mAP@50	mAP@50–95				Params (M)	GFLOPs	Latency (ms)
		All	Small	Medium	Large			
YOLOv8n (CIoU baseline)	95.89	75.12	22.01	67.04	80.12	3.01	8.1	1.67
+ EIoU (loss level)	96.45	75.46	23.34	68.16	79.71	3.01	8.1	1.74
EDCS-YOLOv8 (full)	96.17	**75.78**	**23.72**	**69.04**	**80.15**	3.46	9.0	2.41

**Table 2:** Seed replicates of the three main configurations (size-stratified mAP@50–95, %). Mean ± standard deviation over seeds 0, 1, and 42. The best value in each column is shown in bold.

Configuration	All, Per Seed			Mean ± Std			
	s0	s1	s42	All	Small	Medium	Large
CIoU baseline	75.12	75.08	75.08	75.09 ± 0.02	22.95 ± 1.31	67.73 ± 0.60	79.36 ± 0.67
+ EIoU	75.46	74.87	75.07	75.13 ± 0.30	23.99 ± 0.90	67.92 ± 0.21	79.54 ± 0.29
EDCS-YOLOv8 (full)	75.78	75.11	75.82	**75.57 ± 0.40**	23.59 ± 0.26	**68.88 ± 0.35**	**79.96 ± 0.37**

**Table 3:** Image-level paired bootstrap (B=5000 iterations; in each iteration the same resampled test set is evaluated for seeds 0, 1, and 42, and the three per-seed differences are averaged). Δ is the bootstrap mean of the three-seed mean mAP@50–95 difference (points); P(Δ>0) is the bootstrap proportion of positive differences.

Comparison	Δ (Pooled Mean)	95% CI	P(Δ>0)
EDCS − CIoU	+0.46	[+0.16,+0.77]	0.999
EDCS − EIoU	+0.43	[+0.12,+0.72]	0.996
EIoU − CIoU	+0.04	[−0.25,+0.31]	0.601

**Table 4:** Controlled search over five three-module combinations (all on EIoU, test mAP@50–95, %). Acceptance: small and medium improve and large regresses by at most 0.5. The best value in each column is shown in bold.

Group	Axis	All	Small	Medium	Large
G1 (ours)	elongated geometry	**75.78**	23.72	**69.04**	80.15
G4	upsampling fusion	75.74	**24.56**	68.38	**80.67**
G2	large receptive field	75.68	23.44	68.97	80.13
EIoU baseline		75.46	23.34	68.16	79.71
G3	lightweight attention	74.71	23.68	67.61	78.54
G5	cross-axis all-star	74.18	22.25	66.91	77.67

**Table 5:** Regression-loss ablation: size-stratified mAP@50–95 (%) with identical architecture. The best value in each column is shown in bold.

Loss	All	Small	Medium	Large
CIoU (baseline)	75.12	22.01	67.04	80.12
SIoU [59]	75.39	22.20	**68.23**	80.09
WIoU [60]	75.39	**24.55**	67.71	80.06
EIoU [37]	**75.46**	23.34	68.16	79.71

**Table 6:** Loss–architecture factor grid (test mAP@50–95, %, seed 0). The CIoU row of the EDCS architecture completes the 2×2 design. The best value in each column is shown in bold.

	All	Small	Medium	Large
CIoU, baseline architecture	75.12	22.01	67.04	80.12
EIoU, baseline architecture	75.46	23.34	68.16	79.71
CIoU, EDCS architecture	75.63	24.21	68.61	80.02
EIoU, EDCS architecture	**75.78**	**23.72**	**69.04**	**80.15**

**Table 7:** Eight-configuration ablation grid on top of the architecture (size-stratified mAP@50–95, %). D = dynamic snake convolution (P5), S = strip-gated block (neck-P3), C = context anchor attention (SPPF). Δ is the change in overall mAP@50–95 relative to the CIoU baseline (first row). A checkmark (✓) denotes an enabled component; a cross (×) denotes a disabled one. The best value in each column is shown in bold.

EIoU	D	S	C	All	Δ	Small	Medium	Large	Params (M)	Latency (ms)
×				75.12	–	22.01	67.04	80.12	3.01	1.67
✓				75.46	+0.34	23.34	68.16	79.71	3.01	1.74
✓	✓			75.30	+0.18	23.70	68.43	79.59	3.27	2.12
✓		✓		75.43	+0.31	**25.47**	68.57	79.60	3.07	2.23
✓			✓	75.48	+0.36	22.51	68.15	80.08	3.15	1.72
✓		✓	✓	73.47	−1.65	23.09	68.13	77.63	3.20	2.39
✓	✓	✓		74.86	−0.26	23.56	68.25	78.51	3.33	2.48
✓	✓		✓	75.28	+0.16	23.52	68.11	79.66	3.41	2.07
✓	✓	✓	✓	**75.78**	**+0.66**	23.72	**69.04**	**80.15**	3.46	2.41

**Table 8:** Aspect-ratio terciles of the SeaShips test set (mAP@50–95, %; subset accounting, see text). Areas are at the 640 evaluation scale; bold marks the best result within each tercile. The best value in each column is shown in bold.

AR Tercile	*n*	Median AR	Mean Area (px^2^)	CIoU	EIoU	EDCS	Δ (EDCS−CIoU)
low (<3.92)	1561	3.07	10,864	**71.33**	71.26	71.06	−0.28
mid (3.92–5.66)	1561	4.68	13,665	70.21	70.21	**71.04**	+0.83
high (≥5.66)	1561	7.17	15,532	62.51	63.47	**65.85**	+3.34

**Table 9:** Within-class control: each class is split at its own AR median; Δ is the EDCS−CIoU difference in subset mAP@50–95 (points). Mean areas (px^2^, 640 scale) document the residual scale difference between the halves; the final row gives the unweighted mean over the six classes.

Class	Split AR	*n* (Low/High)	Mean Area (px^2^)		Δ Low-AR Half	Δ High-AR Half
			Low	High		
ore carrier	5.5	497/498	16,055	21,420	+1.08	+1.86
bulk cargo carrier	4.0	218/219	14,726	10,092	−0.86	+1.42
general cargo ship	3.8	594/594	5753	4731	−0.64	+0.92
container ship	4.6	372/372	22,434	20,181	+0.64	+1.49
fishing boat	7.6	535/536	12,943	13,545	+1.01	+1.85
passenger ship	3.0	124/124	8539	9226	+0.25	+1.67
mean	–	–	–	–	+0.25	+1.53

**Table 10:** Size-stratified results for the tested small-object modifications and EDCS-YOLOv8 (test mAP@50–95 deltas vs. CIoU baseline). A dash indicates that no separate size-stratified value is available.

Modification	Small Δ	Large Δ
+ P2 detection head	+2.58	−5.03
+ coordinate attention (P3)	+0.95	−2.77
+ decoupled regression head	–	(−1.71 All)
+ lightweight-attention group (G3)	+1.67	−1.58
+ EIoU (loss level)	+1.33	−0.41
EDCS-YOLOv8 (full)	+1.71	+0.03

**Table 11:** Comparison with ship-specific detectors on SeaShips. Literature numbers are as reported by the original papers and are not protocol-matched (splits, base scales, and hardware differ; EGM-YOLOv8 uses a YOLOv8l base and an 8:1:1 random split; ours uses YOLOv8n on the official split). Our latency is the forward pass at batch 1/FP32/640 on an RTX 4060 Laptop; the FPS value is forward-only (inverse latency) and excludes pre-/post-processing. The best value in each column is shown in bold.

Method	mAP@50	mAP@50–95	Params (M)	FPS
AARN [23]	0.947	0.702	35.82	45
YOLOv5ship [19]	0.976	0.710	40.30	60
IL-YOLOv5 [20]	0.989	0.790	29.80	94
ALF-YOLO [24]	**0.991**	**0.850**	42.51	38
EGM-YOLOv8 [25]	**0.991**	0.848	38.40	45
EDCS-YOLOv8 (ours)	0.962	0.758	**3.46**	**≈415**

**Table 12:** Cross-dataset evaluation on the WUTDet Part A test split (size-stratified mAP@50–95, %; single seed). The large bucket contains only 179 instances and is correspondingly noisy. The best value in each column is shown in bold.

Configuration	All	Small	Medium	Large
YOLOv8n (CIoU baseline)	49.86	39.68	65.62	**87.55**
+ EIoU	**50.20**	**40.16**	**65.82**	86.64
EDCS-YOLOv8 (full)	49.97	40.04	65.67	86.12

**Table 13:** Per-class AP@50-95 with support counts on the SeaShips test set (%). The best value in each column is shown in bold.

Class	Support	CIoU	EIoU	EDCS	Δ (EDCS−CIoU)
ore carrier	995	76.58	77.65	**78.08**	+1.50
bulk cargo carrier	437	81.13	80.62	**81.31**	+0.18
general cargo ship	1188	69.43	**69.50**	69.19	−0.24
container ship	744	78.12	78.33	**79.07**	+0.95
fishing boat	1071	75.29	75.56	**76.29**	+1.00
passenger ship	248	70.19	**71.09**	70.76	+0.57

## Data Availability

The SeaShips and WUTDet Part A datasets used in this study are publicly available. The evaluation, statistical-analysis, and warm-start scripts are publicly available at https://github.com/CarryJohn0411/edcs-yolov8 (accessed on 27 October 2026).

## Abbreviations

The following abbreviations are used in this manuscript:

AIS	Automatic Identification System
AR	Aspect Ratio
CAA	Context Anchor Attention
CI	Confidence Interval
CIoU	Complete Intersection over Union
CNN	Convolutional Neural Network
DFL	Distribution Focal Loss
DSConv	Dynamic Snake Convolution
EIoU	Efficient Intersection over Union
FPS	Frames Per Second
GFLOPs	Giga Floating-Point Operations Per Second
mAP	mean Average Precision
NMS	Non-Maximum Suppression
SGD	Stochastic Gradient Descent
SPPF	Spatial Pyramid Pooling–Fast

## Appendix A. Validation-Split Selection Record

Table A1 lists the best validation mAP@50–95 (1750-image validation split) of every trained configuration, as recorded in the training-time validation curves of each run. The validation ordering coincides with every selection reported in the paper: EIoU ranks first among the four losses (77.44 vs. 77.13 for CIoU), the elongated-geometry group G1 ranks first among the five module groups (77.98 vs. 77.62 for the runner-up G4), and the complete configuration ranks first in the eight-configuration grid (77.98 vs. 77.59 for +S). The same design decisions would therefore have been reached using the validation split alone. The tables in the main text report test numbers for the selected configurations, as stated in Section 4.3.

**Table A1:** Best validation mAP@50–95 for the fifteen original configurations (no test annotations involved). The best value in each column is shown in bold.

Role	Configuration	Val mAP@50–95
baseline	CIoU	77.13
loss search	SIoU	77.36
	WIoU	77.30
	**EIoU (selected)**	**77.44**
group search	G2, large receptive field	77.38
	G3, lightweight attention	77.08
	G4, upsampling fusion	77.62
	G5, cross-axis combination	76.86
	**G1, elongated geometry (selected)**	**77.98**
ablation grid	+D (snake only)	77.57
	+S (strip only)	77.59
	+C (CAA only)	77.20
	D+S	77.10
	D+C	77.46
	S+C	75.93

## Appendix B. Warm-Start Loading Report

The warm-start script records, for every tensor of the final EDCS-YOLOv8 model, how it is initialized from the COCO-pretrained YOLOv8n checkpoint (official Ultralytics release). Table A2 summarizes the categories; the counts cover all 437 state-dict entries and account for the 3,464,150 trainable parameters of the model (3.46M as reported in the main text; batch-normalization statistics buffers are excluded from the counts). The 151 unused checkpoint tensors all belong to the 80-class COCO detection head and are replaced by the 6-class SeaShips head, as expected.

**Table A2:** Loading report of the final model from the COCO-pretrained YOLOv8n checkpoint. The snake convolution’s main branch is included in the index-remapped category; the last three rows break down the newly initialized tensors. Counts refer to trainable parameters; batch-normalization statistics buffers stored in the state dict are excluded.

Transfer Category	Tensors	Parameters
copied by exact name match	204	1,310,416
index-remapped after the neck insertion	145	1,553,890
newly initialized (near-identity scheme)	88	599,844
of which: StripBlock (P3 neck)	34	52,992
of which: DSConv offset and projection branches	38	408,612
of which: CAA attention branch	16	138,240
total	437	3,464,150
